# Students' Perceptions on an Interprofessional Ward Round Training – A Qualitative Pilot Study

**DOI:** 10.3205/zma001013

**Published:** 2016-04-29

**Authors:** C. Nikendei, D. Huhn, G. Pittius, Y. Trost, T. J. Bugaj, A. Koechel, J.-H. Schultz

**Affiliations:** 1 University of Heidelberg Medical Hospital, Department of General Internal and Psychosomatic Medicine, Heidelberg, Germany; 2 Louise von Marillac-School for Health Professions, Heidelberg, Germany; 3 IB-GIS mbH Medical Academy for Physiotherapy, Mannheim, Germany

**Keywords:** medical education, ward round training, medical students, nursing students, physiotherapy students, standardised patients

## Abstract

**Introduction: **Ward rounds are an essential activity for interprofessional teams in hospital settings and represent complex tasks requiring not only medical knowledge but also communication skills, clinical technical skills, patient management skills and team-work skills. The present study aimed to analyse final year students’, nurses’ as well as physiotherapists’ views on a simulation-based interprofessional ward round training.

**Methods: **In two successive passes a total number of 29 final year students, nursing students and physiotherapy students (16 in the first run, 13 in the second) volunteered to participate in two standardized patient ward round scenarios: (1) patient with myocardial infarction, and (2) patient with poorly controlled diabetes. Views on the interprofessional ward round training were assessed using focus groups.

**Results: **Focus group based feedback contained two main categories (A) ward round training benefits and (B) difficulties. Positive aspects enfolded course preparation, setting of the training, the involvement of the participants during training and the positive learning atmosphere. Difficulties were seen in the flawed atmosphere and realization of ward rounds in the daily clinical setting with respect to inter-professional aspects, and course benefit for the different professional groups.

**Conclusion: **The presented inter-professional ward round training represents a well received and valuable model of interprofessional learning. Further research should assess its effectiveness, processes of interprofessional interplay and transfer into clinical practice.

## Zusammenfassung

**Einleitung: **Visiten stellen eine relevante und zentrale Tätigkeit interprofessioneller Teams im Klinikalltag dar und repräsentieren dabei eine komplexe Aufgabe, welche nicht nur medizinische Kenntnisse erfordert, sondern auch kommunikative und klinisch-technische Fertigkeiten sowie patientenmanagement- und teambezogene Kompetenzen. Die vorliegende Studie hatte zum Ziel, die Gesichtspunkte von Medizinstudierenden, Krankenpflegeschülern und Physiotherapieschülern hinsichtlich eines simulationsbasierten interprofessionellen Visitentrainings zu analysieren.

**Methoden:** In zwei aufeinander folgenden Durchgängen nahmen insgesamt 29 Medizinstudierende, Krankenpflege- und Physiotherapieschüler (16 im ersten Durchgang, 13 im zweiten) an zwei Visitenszenarien mit standardisierten Patienten teil: (1) Patient mit Myokardinfarkt und (2) Patient mit schlecht eingestelltem Diabetes. Die Evaluation des interprofessionellen Visitentrainings erfolgte mit Hilfe von Fokusgruppeninterviews.

**Ergebnisse: **Die Resultate der Fokusgruppen ließen sich in die beiden Hauptkategorien (A) Nutzen des Visitentrainings sowie (B) Schwierigkeiten mit dem Visitentraining differenzieren. Als positive Gesichtspunkte wurden dabei die erfolgte klinische Vorbereitung durch das Training, das Setting, in dem es stattfand, das Engagement der Teilnehmer sowie die positive Lernatmosphäre benannt. Schwierigkeiten wurden im mangelnden Realitätsbezug des Trainings bzgl. interprofessioneller Aspekte sowie im unterschiedlichen Nutzen des Trainings für die einzelnen Berufsgruppen gesehen.

**Fazit: **Das beschriebene interprofessionelle Visitentraining wurde von den Teilnehmern gut angenommen und kann als wertvolles Modell für interprofessionelles Lernen dienen. Zukünftige Forschung sollte die Effektivität, die Prozesse des interprofessionellen Zusammenspiels sowie den Transfer in die klinische Praxis fokussieren.

## Introduction

Ward rounds are an essential duty for doctors within hospital settings [21]. Conducting ward rounds represents a complex task requiring proficiency not only in medical knowledge but also in communication skills, clinical technical skills, patient management skills and team-work skills. Wray et al. [32] investigated ward round characteristics based on observations of house staff teams with upper-level residents revealing that the average daily time spent on a single patient visit amounts to 4.6 minutes. This short period of time available per patient necessitates that the involved team must be operating highly effective. For tomorrow’s doctors, however, severe deficits in conducting ward rounds could be detected with predominant insufficiencies in reviewing charts and initiating appropriate prescriptions and documentation [20]. Furthermore, within ward rounds nurses’ knowledge is under-represented as they contribute much less medical information than doctors [30]. Besides, a study by Montague and Hussain [17] showed that one-third of patients perceived staff to use language during ward rounds that was difficult to understand suggesting insufficient communication in general. 

The role of interprofessional communication and collaboration in health care is becoming increasingly important [1], [33]. Interprofessional collaboration can be defined as an integrative cooperation of different health professionals, blending complementary competences and skills, thus allowing the best use of resources [25]. Major national organizations such as the Interprofessional Education Collaborative in the US [11] and the Canadian Interprofessional Health Collaborative [3] postulated more competencies for health professionals in areas such as interprofessional teamwork and communication recently. Recent discussions [33] indicate that already students respectively trainees in medical professions need interprofessional competencies necessarily for their future clinical practice. Particularly in the English-speaking world interprofessional learning has already been integrated into curricula [27]. So far, teaching methods of interprofessional learning are mostly studied in the context of small group settings, case analyses or simulations [28], [23], [18], however models of inter-professional ward round trainings are rare.

Recently, an innovative model for ward round training using standardized patients (SP) was introduced in final year medical education. [19]. Hereby, final year students assumed the role of either doctor, nurse or final year student with role-specific instructions and provided each other with peer-feedback during the training session. A first model not only integrating final year students but also nursing students was introduced by Pederson et al. [22]. Here, medical students and nursing students participate in ward round trainings with a focus on discharge planning.

In this short report, we aimed to analyse final year students’, nursing students’ as well as physiotherapist students’ views on an interprofessional ward round training. The training was based on a context-based ward round scenario with the aim of an interprofessional exchange regarding occupation specific treatment goals as well as mutual treatment planning. Therefore an interprofessional ward round training with standardized patients enfolding two different scenarios was designed on bases of the model developed by Nikendei et al. [19]. Clinical scenarios were: ward round with

1. a patient with myocardial infarction, and
2. a patient with poorly controlled diabetes.

Participants took part in a subsequent focus group analysis. The aim of the focus group interviews was to explore students’ perceptions of their experiences during the interprofessional ward round training.

## Methods

### Sample

In two successive passes a total number of 29 students (16 in the first run, 13 in the second) volunteered to participate in the study. Of these, 13 (45%) were final year medical students at Heidelberg University Medical Hospital (8 female, 5 male) with a mean age of 26.7 years, 9 (31%) were final year nursing students at Louise von Marillac-School for Health Professions, Heidelberg (7 female, 2 male) with a mean age of 21.9 years and 7 (24%) were physiotherapy students at IB-GIS mbH Medical Academy for Physiotherapy, Mannheim (4 female, 3 male) with a mean age of 23.6 years.

#### Study design

The two scenario ward round training sessions were embedded into a mini-curriculum of 4 interprofessional learning units of 240 min each: social gathering + PBL-case-analysis, basic life support training, interprofessional ward round training, SP-based communication training. All units were designed by an interprofessional teaching team consisting of medical doctors (CN, JS and AK), nurses (GP), physiotherapists (YT), and research assistants (DH). Learning objectives were discussed according to the Basel Consensus Statement "Communicative and Social Competencies in Medical Education" [13].

The ward around training proceeded as follows: Groups of three students each participated in two different standardized patient ward round scenarios [2]:

1. 50 year old, stout Mr. Behrens with stent implantation following myocardial infarction,
2. 45 year old Mrs. Vogel with diabetes-related compliance problems, high blood pressure and leg ulcer.

The three students assumed the role of doctor, nurse or physiotherapist according to their own profession and received role-specific instructions. Doctors’ tasks involved gaining an overview of the patient case, related patient files and patient medication charts. Doctors were further required to define consultation goals, conduct the ward round consultation and subsequently re-evaluate the patient’s therapy. The re-evaluation included completion of medication charts, provision of instructions for the nursing staff and the preparation of written notes regarding the proposed management. Nurses’ tasks involved bringing questions about patients’ mobilisation and activation into the ward as well as talking about patients’ discharge management. Due to their more personal relationship to patients they also played an important role as crucial link between patients’ demands and wishes on the one hand and doctors’ decisions on the other hand. The physiotherapists were asked to bring patients’ physical activities into the wards. They were to suggest specific measures like ergometer trainings or walking units. Ward round sessions were followed by an instructor-guided debriefing and feedback round in which teachers of all health professions were actively engaged.

#### Focus group interviews

A qualitative approach is not only appropriate for exploring what students are thinking but also why they are thinking it [15]. Focus group discussions in peer groups offer a relatively safe environment in which the power imbalance between researcher and subjects is mitigated. Group discussions foster the deeper analysis of perceptions of a topic and may even refine or modify them. Hence, focus group interviews seem to be appropriate for the present research issue.

An experienced moderator actively encouraged input from all participants and ensured that different opinions could be expressed. The students were asked to note their thoughts on a topic before these were discussed in the group. Each ward round training was followed by a focus group session which resulted in four interviews in total, lasting approximately 20 minutes each. The sessions were moderated by one of the authors (DH). In order to ensure consistency across groups, an interview guide was used for the interviews [15] (see Table 1 (Tab. 1)). During the group discussions the assistant moderator (TB) took comprehensive notes and video documented the sessions. The videotapes were transcribed verbatim. Discussion summary reports were sent to the students for approval.

#### Qualitative content analysis

The verbatim-transcribed discussions were analyzed via qualitative content analysis using the software MaxQDA (version 11, VERBI GmbH, Berlin). In accordance with guidelines for qualitative inductive content analysis [16] thematic categories were not predefined, but were developed from transcript content. Therefore, an open coding of all discussions was conducted first to search for recurring topics. Single or multiple sentences were identified as a code, representing the most elemental unit of meaning [26]. Next, the codes were summarized into relevant themes for each participant. As themes recurred across participants, they were then compared and adapted until a number of relevant themes for all participants could be defined. The assignment of codes to specific themes was conducted by two independent analysts (CN and JS), discussed to reach consensus and adjusted if necessary. In the final step, themes were consolidated into two relevant categories.

## Results

### Main categories and themes resulting from qualitative analysis

The qualitative analysis of the transcripts identified 45 relevant single tutees’ statements. From these statements, nine themes and two main categories were derived. The main categories included (A) ward round training benefits and (B) ward round training difficulties. These main categories contained four themes each (i.e. A.1 to A.4).

#### Definition of categories

In the following, we provide definitions for the main categories. 

##### (A) Ward round training benefits

This category describes different positive and beneficial experiences participants reported concerning the ward round training. The category includes four relevant themes. The theme (A.1) “Improved understanding of the situation, tasks and common objective” envelopes students’ sustainable experiences of other professional groups as well as their tasks, an improvement in the understanding of the task distribution between the different professional groups and the experience of the professional groups’ mutual support considered as beneficial for the patients’ overall treatment. (A.2) “Ward round practice” describes the beneficial experience of understanding the basic ward round sequence as well as the individual role and behaviour in a realistic setting. The theme (A.3) “Involvement of all professional groups” includes students’ feelings of interprofessional action and care given through the fact that all professional groups were equally involved during ward round training. The fact that ward round training was held in casual attire and evaluations took place without pressure was perceived to contribute to the relaxed and informal tone of the setting, summarized under the theme (A.4) “Relaxed learning atmosphere”. For more details see Table 2 (Tab. 2).

##### (B) Ward round training difficulties

The second category highlights students’ critical aspects concerning the ward round training. Five relevant themes emerged. The theme (B.1) “Ward round training versus reality” includes the participants’ perception that the training was unrealistic as usual everyday stresses were omitted and all professional groups were present, which is rarely the case during genuine ward rounds. (B.2) “Ward rounds: a flawed starting point for inter-professional action” describes the students’ experience that ward rounds generally failed to offer the best opportunity for inter-professional learning and action since the subsequent situations after rounds were of more interest. The theme (B.3) “Unfavourable time management” summarises the participants’ statements that ward round preparation had received too much attention, while time at bedside had been neglected. Finally, (B.4) “Lack of equal benefit” includes students’ criticism concerning the fact that the training did not provide all professional groups with equal learning benefit; in these opinions, nurses and physiotherapists had not been able to gain the same added value from the training. More detailed information is given in Table 3 (Tab. 3).

#### Similarities between the professional groups

The three professional groups mostly agreed that the training allowed them to learn a great deal from each other and could be a potential benefit to patients as well. They liked the course’s atmosphere and perceived themselves as being part of a valuable interdisciplinary learning opportunity. However, the groups were also in agreement in voicing criticism that the training was too artificial and distant from reality.

#### Divergences between the professional groups

The groups mostly differed in the aspect of how beneficial a ward round practice was to them: While medical students appreciated the scenario, since they had never practiced ward rounds before, nursing students where already quite familiar with this format; and physiotherapy students indicated they would not go on ward rounds in real life anyway. Nursing and physiotherapy students also complained about the project’s focus on medicine, with medical students performing the majority of the tasks.

## Discussion

Our study describes an interprofessional ward round training for final year medical, nursing and physiotherapy students and its evaluation using focus groups. The qualitative analysis of the focus group interviews revealed that the training was well received by participants and proved a valuable source of interdisciplinary learning. Students criticized the ward round simulations’ degree of realism, time management and the fact that the training failed to benefit all participating professions equally.

The training was perceived as a valuable resource enabling participants to learn from the other professions’ perspectives, their occupation related responsibilities, task distribution as well as diagnostic and therapeutic procedures. The patient was experienced as being a common and combining element resulting in a feeling of team coherence. Although the lack of equal involvement of all three professional groups was criticized and suggestions regarding other scenarios for interprofessional team work were made, our ward round training represents a valuable opportunity to introduce students to ward round sequence and procedures as well as to the required interprofessional team work. This seems especially important, as ward rounds in particular continue to be characterized by suboptimal supervision and the trainees’ insufficient specific guidance [8].

From a methodological point of view, participants perceived ward round training as rather artificial and idealized. Furthermore, time management was criticized and participants felt that the lacking possibility to equally involve all three professional groups in the role-play instructions and tasks was unfavorable. Although standardized patients are perceived to portray a high degree of realism [7], the training situation was not seen to be realistic enough. Regarding simulations’ degrees of realism, there are contradicting results to whether the simulations’ realism is related to its effectiveness [12], [6].

The participants criticized the “lack of an equal benefit” concerning the different professional groups. To be of equal benefit to all professional groups, the relevance for the future professional practice of each discipline must be recognizable in all learning subunits [31]. This might be more easily realized in joint new conceptions of courses than in primarily mono-professionally designed trainings as done in this project. This is of specific importance as ward rounds are considered to be the most central interface of inter-professional collaboration in hospitals [14]. “Ward-based interprofessional clinical settings offer students realistic experiences for ideal clinical 'trying on' a professional role: 'experiencing independence and autonomy'; 'seeing clearly what the own profession is all about'; 'altered images of other professions'; 'ways of communicating and collaborating' and 'becoming a functioning team' [9]. To perform a truly interprofessional and not merely a multiprofessional training, a well-guided, realistic scenario that involves all addressed groups of learners is needed. Furthermore, interprofessional key aspects should be addressed and discussed prior and following the ward-based training and should be jointly reflected by the different participating representatives.

Nevertheless, we are convinced that these interventions represent an important step towards students’ improved preparation for on-ward clinical practice and in turn towards the further assurance of patients’ safety. Further research should investigate interprofessional teams’ ward round skills in a real life setting. As this is the first interprofessional ward round training of its kind to be performed in our faculty, criticism of insufficient time management and weighing of roles must be taken in to account and amended for further implementations. Especially, nursing and physiotherapy students must become more involved within the scenarios in future.

## Limitations

There are several limitations regarding our study. The number of participants was limited and objective measures of success are missing. Besides, the qualitative content analysis revealed only two main categories, the benefits and difficulties of the ward round training, both issues could be derived from the interview guide. Another point of criticism is the fact that only the overall results could be presented due to the small amount of data; the two sub items, similarities and divergences between the subitems groups, are of only an exemplary nature. Furthermore, the current study focusses on medical student education. Further research is needed to assess whether gained insights can be transferred to postgraduate ward-round training. The use of in-depth focus group interviews and quotations enabled us to develop a comprehensive picture of this multi-layered topic and led to a number of new aspects regarding interprofessional training. 

## Conclusions

Our interprofessional ward round training represents a well received and valuable model of interprofessional learning. Further research should assess its effectiveness, processes of interprofessional interplay and transfer into clinical practice.

## Funding

This study was supported by the Ministry for Science, Research and Art Baden-Württemberg, Germany, project: „Assurance of academic success in high-risk groups – study for the improvement of intercultural communication“; project identification number: D 100011720; AZ32-402.17(05)/34.

## Competing interests

The authors declare that they have no competing interests.

## Figures and Tables

**Table 1:**
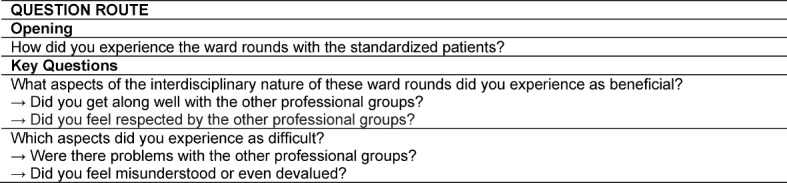
Questioning route for focus group interviews

**Table 2:**
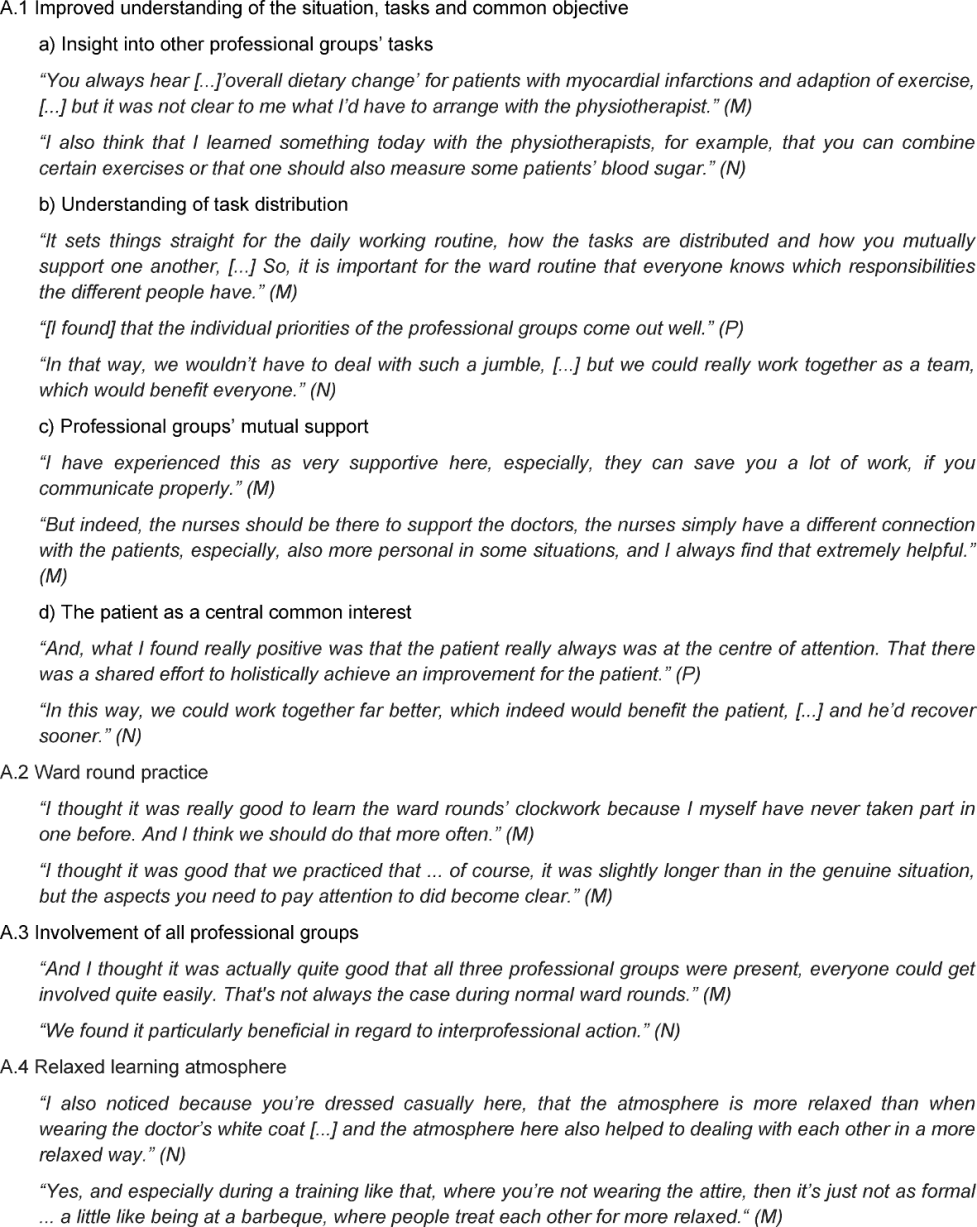
Main Category A – Ward round training benefits (M=medical student, N=nursing student, P=physiotherapy student)

**Table 3:**
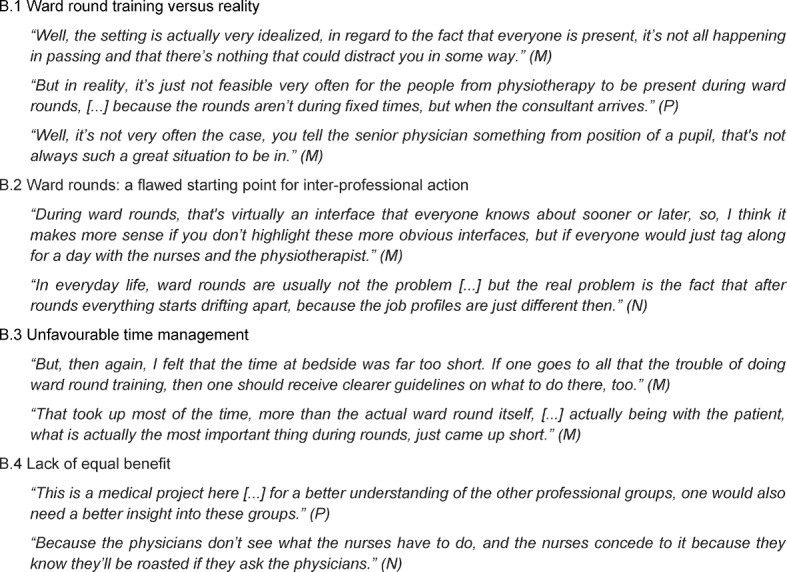
Main Category B – Ward round training difficulties (M=medical student, N=nursing student, P=physiotherapy student)
